# Evaluation of the nation-wide implementation of ALS home monitoring & coaching: an e-health innovation for personalized care for patients with motor neuron disease

**DOI:** 10.1186/s12913-022-08724-6

**Published:** 2022-11-22

**Authors:** M. L. Dontje, E. Kruitwagen-van Reenen, E. van Wijk, E. Baars, J. M. A. Visser-Meily, A. Beelen, on behalf of the Study Group ALS Home monitoring & Coaching, Jim van Os, Leonard van den Berg, Conny van der Meijden, Vincent Cornelissen, Marietta Eimers, Anja Horemans, Esther Kruitwagen, Janneke Sterk, Laura Peeters, Evelien Pirard, Trees Spendel, Anne-Wil Koopman, Remco Timmermans, Germaine Kramer, Evert Schouten, Rineke Jaspers Focks

**Affiliations:** 1 Department of Rehabilitation, Physical Therapy Science and Sports, UMC Utrecht Brain Centre, University Medical Centre, Utrecht, the Netherlands; 2 Centre of Excellence for Rehabilitation Medicine, UMC Utrecht Brain Centre, University Medical Centre Utrecht, and De Hoogstraat Rehabilitation, Utrecht, the Netherlands; 3 Department of Rehabilitation, Northwest Clinics, Alkmaar/Den Helder, Alkmaar, The Netherlands; 4 De Vogellanden centre for rehabilitation, Zwolle, The Netherlands

**Keywords:** Motor neuron disease, Amyotrophic lateral sclerosis, Rehabilitation, Telemedicine, Technology, eHealth, Implementation, Evaluation

## Abstract

**Background:**

To improve the care for patients with motor neuron disease an e-health innovation for continuous monitoring of disease progression and patients’ well-being (ALS H&C) was implemented in 10 multidisciplinary rehabilitation settings. The first aim was to evaluate the implementation of ALS H&C by assessing several implementation outcomes, technology acceptance and usability of the innovation according to the end users. The secondary aim was to explore differences in these outcomes between the teams with sustainable and unsustainable implementation.

**Methods:**

The chosen implementation strategy was a combination of the implementation process model by Grol & Wensing and a participatory action research approach. In three meetings with multidisciplinary project groups the innovation was introduced, the expected barriers/facilitators identified, and action plans to resolve each barrier developed. After a 3-month pilot phase, patients and their healthcare providers were asked to complete an online evaluation survey to assess implementation outcomes, based on Proctor’s evaluation framework (i.e., acceptability, feasibility, fidelity, sustainability). Telemedicine technology acceptance was assessed according the technology acceptance model of Chau, and user experiences with the System Usability Scale (SUS). Implementation outcomes of teams with sustainable implementation (continuation after completion of the pilot phase) and unsustainable implementation (discontinuation after the pilot phase) were compared.

**Results:**

The implementation outcomes from the patients’ perspective (*N* = 71) were positive; they found ALS H&C to be an acceptable and feasible care concept. Patients’ technology acceptance was high, with positive attitudes towards ALS H&C, and positive views on perceived technology control, usefulness, and ease of use. Patients rated their satisfaction with the (web) app on a scale from 1 (not satisfied at all) to 10 (very satisfied) with a 7.0 (median; IQR 1.0). Healthcare providers (*N* = 76) also found ALS H&C acceptable and appropriate as well, but were less positive about the feasibility and usability of ALS H&C (mean SUS 58.8 [SD 11.3]). ALS H&C has largely been implemented as intended and the implementation was sustainable in 7 teams. Teams who discontinued ALS H&C after the pilot phase (*N* = 2) had more fidelity issues.

**Conclusions:**

A participatory action research approach supported by theoretical approaches used in implementation science led to a sustainable implementation of ALS H&C in 7 of the participating teams. To improve implementation success, additional implementation strategies to increase feasibility, usability and fidelity are necessary.

**Trial registration:**

Trial NL8542 registered at Netherlands Trial Register (trialregister.nl) on 15th April 2020.

**Supplementary Information:**

The online version contains supplementary material available at 10.1186/s12913-022-08724-6.

## Background

Amyotrophic lateral sclerosis (ALS) is a rare progressive neurodegenerative disease that affects only two to three people per 100,000 people per year [1, 2]. There is no effective cure and most patients die within 3–4 years of the first symptoms occurring [1, 2]. Multidisciplinary care is the mainstay of treatment, focusing on optimizing quality of life and prolonging survival [3–5]. According to international guidelines, patients with ALS should be monitored by a multidisciplinary care team at regular intervals (e.g. NICE-MND guideline recommends every 2–3 months) [5–8]. Due to the complex and highly variable disease process, a fixed frequency may not meet the needs of all people with ALS in all phases of their disease. In order to provide proactive, tailored care, it is imperative to monitor patients’ well-being and disease progression closely, also between outpatient visits [5, 7, 8]. E-health innovations, that can continuously monitor disease progression at home, thereby reducing the burden for patients and their caregivers, have great potential [9–11].

We, therefore, developed the e-health innovation ‘ALS Home-monitoring and Coaching’ (ALS H&C) in the ALS clinic of the University Medical Centre Utrecht (expert centre for ALS), the Netherlands. With ALS H&C, patients can be monitored continuously and remotely by the ALS care team, because patients pass on measurements (data) regarding their well-being (daily), body weight (weekly) and functional status (monthly) using a mobile application [9, 12]. ALS H&C was well received by patients and healthcare professionals in the initial setting [9], where it has been fully integrated into the usual care for patients with ALS since 2017 [13]. Given the positive experiences and the significant added value for patients [9, 13], it is paramount to make this new concept of ALS care available for all patients with ALS in the Netherlands. To achieve this, ALS H&C should be implemented in all ALS care teams in the Netherlands.

Implementing a new care concept can be challenging, especially in a multidisciplinary setting, because it requires organizational changes as well as behavioral changes of numerous people. Implementation efforts frequently rely on ‘letting it happen’ and ‘helping it to happen’ approaches, but research has shown that ‘making it happen’ strategies are much more likely to succeed [14, 15]. In other words, diffusion or dissemination of information regarding ALS H&C alone would most likely not lead to a successful implementation, but purposeful, active and persistent support during implementation probably would. A strong theoretical foundation in implementation science is imperative when designing an implementation project [16–19]. Process models can give practical guidance regarding the steps necessary to improve the likelihood that an innovation will be integrated into daily clinical practice [16]. Determinant frameworks are useful for establishing a comprehensive view of determinants (factors that can strengthen or weaken the impact of implementation strategies and the effectiveness of the implemented innovations) [16]. This in turn can guide the selection of behaviour change strategies that can target potential barriers for implementation [16]. To comprehensively evaluate the success of implementation efforts, specific evaluation frameworks are recommended. These should address relevant aspects of implementation, such as acceptability, adoption, appropriateness, feasibility, fidelity, implementation cost, penetration, and sustainability [16, 20].

In addition, research has shown that the inclusion of key stakeholders is an important determinant for implementation success [21–23]. Key stakeholders, participating as co-researchers rather than subjects, is one of the core elements of participatory action research. This aims to simultaneously understand and change a current situation, with researchers and the people who will be affected by the intended changes working together [23, 24]. Many people will be affected by the implementation of ALS H&C, including physiatrists, allied health professionals, managers, planners, patients and their informal caregivers. Involvement of these key stakeholders will ensure that their unique perspectives on the matter will be considered during the implementation process.

Others have argued that the combination of implementation science and action research approaches is a promising method for integrating research findings into practice in a sustainable way [23, 25]. The combination of participatory action research and implementation science has been used successfully before [26, 27]. To our knowledge, this approach has not yet been used for implementing an e-health innovation in a multidisciplinary setting.

The first aim was to evaluate the implementation of ALS H&C in multidisciplinary rehabilitation settings, using the above-mentioned combined approach. We assessed several implementation outcomes, the technology acceptance, and the usability of the innovation, according to the end users. The secondary aim was to explore differences in outcomes between the teams where implementation was sustainable and teams that discontinued ALS H&C (unsustainable implementation).

## Methods

### Implementation

The implementation study was carried out between February 2020 and December 2021. The study protocol has been described in more detail previously [12]. In brief, ALS H&C has been implemented in 10 multidisciplinary ALS care teams, selected based on their setting (university hospital, regional hospital, rehabilitation centre), geographical location (urban/rural), and number of patients with motor neuron disease (amyotrophic lateral sclerosis [ALS]/ progressive muscular atrophy [PMA]/ primary lateral sclerosis [PLS]) in order to ensure a wide variety in participating teams. The chosen implementation strategy was a combination of the implementation process model by Grol & Wensing [28] and an exemplarian participatory action research approach, illustrated in supplemental Fig. 1 [29]. The central theme within this approach was the collaboration with the main stakeholders of each participating centre, including the physiatrist(s) of the ALS care team, health care professionals, the manager of the rehabilitation centre or department, planners, and ALS/PMA/PLS patients and informal caregivers. The researchers (AB + MLD) acted as advisors and coordinated the entire process. The implementation strategy consisted of three main phases. In the thematic phase, the researchers had an introductory meeting with the participating teams in which they explained the core elements of ALS H&C. Teams had the opportunity to ask questions and to discuss any potential concerns about implementing and working with ALS H&C. In subsequent focus group meetings, the views, opinions, and expectations of 94 stakeholders regarding implementing ALS H&C within their own setting were discussed. Implementation theoretical determinant frameworks (Grol & Wensing and Theoretical Domain Framework [28, 30]), the expected barriers and facilitators were identified, thematically analyzed, and ordered (REF Dontje et al. barriers/facilitators ALS H&C – recently submitted – will be added as soon as it is accepted for publication). In the crystallization phase, the third meeting took place. During this meeting the teams developed concrete action plans for how to resolve each barrier. A variety of intuitively conceived action plans were developed and put into effect. They were aimed at barriers related to the characteristics of the innovation, characteristics of the individual patients or healthcare provider, and organizational -, and economic context. Action plans included:

- adjusting the healthcare protocol to the context of the location,
- organizing technical support,
- adapting and translating information letters for patients,
- training healthcare coaches to use the platform, developing new work processes, and
- mapping healthcare costs.

In the exemplary phase, teams had 3 months to test and execute their implementation plan and try ALS H&C with 5–10 patients in a pilot study. The final evaluation of the implementation is the focus of the current paper.

### Evaluation of the implementation

At the end of the exemplary phase the participating patients and their healthcare providers were asked to complete an online survey. By then, patients and healthcare providers had approximately 3 months’ experience with ALS H&C. To evaluate the implementation and the user experiences, specific implementation evaluation frameworks were applied [16, 20]. The survey was developed based on the implementation outcomes evaluation framework described by Proctor et al. 2011 [20] and Chau & Hu’s telemedicine technology acceptance model [31]. The System Usability Scale (SUS) was added to assess the usability of the digital platform for healthcare providers [32, 33]. To assess the usability of the app for patients several self-developed questions were added to the survey. App measurements and field notes were also taken into account.

Details of the measurements have been reported [12]. In brief, the following implementation outcomes (Proctor) were assessed: acceptability (e.g., satisfaction), adoption (uptake at organizational and patients’ level), appropriateness/compatibility (e.g., whether it fits within their care needs/the way they work), feasibility (e.g., whether it is suitable for everyday use), fidelity (e.g., whether there were any deviations from the original implementation plans and/or innovation, and patients’ adherence to the agreed-upon monitoring assessments), and sustainability (whether the teams continued with ALS H&C after the completion of the pilot phase). Additional questions on factors related to the (1) individual context (i.e., attitude, perceived technology control), (2) the technological context (i.e., perceived usefulness, perceived ease of use/usability, and (3) the implementation context (i.e., peer influence) were added to the surveys [31]. To assess these factors, most questions (formulated as statements) were answered on a 5-point Likert-scale, ranging from totally disagree to totally agree. Other questions included an overall numerical rating of their satisfaction with the care concept and the mobile application (scale 1- not satisfied at all to 10-very satisfied), statements with a 3-point response format on the actual use of the application (yes, sometimes, no) and on the frequency of measurements (yes, just right, no). The System Usability Scale, which was integrated in the healthcare providers survey, consists of 10 items; answer categories range from 1 - totally disagree to 5 - totally agree. The total score can range from 0 (low usability) to 100 (high usability); a score of 68 is usually considered as ‘average usability’. Patients and healthcare providers were also asked to list the advantages and disadvantages of ALS H&C and whether they intended to keep using ALS H&C within the next 6 months.

### Statistical analyses

The survey data were analyzed using descriptive statistics with IBM SPSS Statistical data software (IBM SPSS Statistics for Windows, Version 26.0. Armonk, NY). All implementation outcomes were assessed from the perspective of patients and healthcare providers separately.

For the ordinal and binary variables, frequencies were calculated and expressed as percentages. For clarity, the answer categories ‘totally agree’ and ‘agree’ were combined as a positive perception on the statement, as were the answer categories ‘totally disagree’ and ‘disagree’ as a negative perception. The normality of continuous variables was tested using Shapiro-Wilk tests. For variables with normal distribution, the descriptive statistics mean values (standard deviation [SD]) were calculated. For not-normally distributed variables, median values (interquartile range [Q3-Q1 = IQR]) were calculated. To assess the subjective usability of the ALS H&C platform according to the healthcare providers, the mean (SD) score on the System Usability Scale (SUS) was calculated.

To assess the fidelity (the degree to which ALS Home-monitoring & Coaching has been implemented in each participating rehabilitation centre as intended [20, 34]), we documented any deviations from 1) the originally planned implementation strategies, and 2) the care protocol for ALS Home-monitoring & Coaching. To assess adherence, which is another part of the fidelity, the following formula was used: (number of completed measurements/ number of agreed upon measurements) * 100%. The median (IQR) adherence was calculated for the three measurements in the e-health application, i.e. well-being, weight, and functioning. The answers to the open-ended questions in the surveys, i.e. the questions related to the advantages and disadvantages of ALS H&C according to the patients and the healthcare providers, were thematically analyzed, taking an inductive approach. Teams were classified as either ‘sustainable implementation’, when the team continued using ALS H&C after completion of the exemplary (pilot) phase, or ‘unsustainable implementation’ when ALS H&C was discontinued after the pilot phase. For further exploration, implementation outcomes of the teams with sustainable implementation were compared to those of the teams with unsustainable implementation. This comparison included a qualitative analysis of fidelity and survey outcomes that were assessed with statements and a quantitative comparison of numeric ratings of acceptability (patients’ and healthcare providers’ ratings of the healthcare concept and the mobile application/platform) and system usability from the healthcare providers.

## Results

### Study sample and response rates

Patient characteristics of all participants are presented in Table 1. At the end of the exemplary phase, 67 of 86 patients (response rate = 77.9%) and 76 of 148 healthcare providers (response rate = 51.4%) completed the questionnaire and provided consent to participate in this evaluation study. Healthcare providers that completed the digital evaluation survey were physiatrists (*n* = 12), physical therapists (*n* = 16), speech therapists (*n* = 10), dieticians (*n* = 6), psychologists (*n* = 2), occupational therapists (*n* = 21), nurse specialists (*n* = 2), physician assistants (*n* = 1), and social workers (*n* = 6). Fourteen of them also had the role of healthcare coach and four the role of knowledge broker. Mean (SD) age was 44.4 (10.2) years; 85.3% were female.

**Table 1:** Patient characteristics

		Patients participating in pilot phase (***N*** = 86)	Patients participating in evaluation (***N*** = 71)
Age (in years)	(median, IQR)	62.0 (14.5)	61.0 (15)
**ALSFRS_R-score at the start of the pilot phase**	(median, IQR)	37 (12)	37 (10)
**Gender**			
**Male**	(n, %)	51 (59.3%)	42 (59.2%)
**Female**	(n, %)	35 (40.7%)	29 (40.8%)
**Diagnosis**			
**ALS**	(n, %)	65 (75.6%)	53 (74.6%)
**PMA**	(n, %)	14 (16.3%)	11 (15.5%)
**PLS**	(n, %)	7 (8.1%)	7 (9.9%)
**Duration of the disease**	(median, IQR)	1 (2)	1 (3)

*ALSFRS-R* Amyotrophic Lateral Sclerosis Functional Rating Scale-revised, ranging from 0 to 48. Patients with higher scores have more physical function, *IQR* Interquartile range, calculated with the formula IQR = Q3-Q1, *n* = sample size

### Implementation outcomes

#### Adoption

Adoption, or the uptake, was assessed at organizational level and at patients’ level. In total, 12 teams were invited, all of them intended to participate in this implementation project (100%). Two teams withdrew before the start due to staffing issues. This resulted in an initial adoption rate at organizational level of 83.3%. One team dropped out before the exemplary (pilot) phase, because their hospital board did not give permission for participation. The main reason for this decision was that the provider of the ALS app was not the hospital’s preferred choice of e-health technology provider. Nine of the 12 teams (75%) completed the full implementation process.

In total, 128 patients were invited to participate in the pilot phase of ALS H&C, of whom 90 agreed to participate. Reasons for not willing to participate included ‘not feeling like it’, ‘too confronting’, and ‘not having enough digital skills’. Three of the 90 patients who agreed to participate did not yet start with ALS H&C at the time of this evaluation, due to specific circumstances; one patient died before he could start. The total number of patients that started with ALS H&C in the pilot phase was 86; thus the adoption rate at patients-level was 67.2%. Three more patients dropped out after the start. The reasons varied from patients preferring (more) personal contact, to patients and their spouses feeling that participating was too much of a burden.

#### Acceptability

Patients were mainly positive about the acceptability of ALS H&C (Table 2, panel A). The majority of the patients (71.7%) was (very) satisfied with the new care concept and the mobile application (68.7%). Patients rated the ALS H&C care concept with an 8.0 (median; IQR 1.0) and the (web) app with a 7.0 (median, IQR 1.0).

**Table 2:** Implementation outcomes – Patients and Healthcare providers

	(Totally) Agree	Neutral	(Totally) Disagree
	%	%	%
Panel A – Patients			
***Acceptability***			
I am satisfied with the care concept ALS H&C	71.7	25.4	3.0
I am satisfied with the ALS H&C app	68.7	23.9	7.5
I find the chat function useful	74.7	11.9	13.5
The healthcare coach usually responds to my messages in a timely manner	88.1	4.5	7.5
I am satisfied with the number of contact moments with the healthcare coach	76.1	20.9	3.0
I like that I can monitor my own measurements (well-being, weight, ALS symptoms)	73.2	23.9	3.0
I find it confronting to monitor my own measurements (well-being, weight, ALS symptoms)	7.5	43.3	49.2
I find the emails I receive to remind me of the measurements annoying	4.5	20.9	74.6
I find the frequency of the emails to remind me of the measurements too high	7.5	80.6	12.0
***Appropriateness/Compatibility***			
I find the care concept ALS H&C fits in well with my care needs	50.7	34.3	14.9
I like that with ALS H&C a healthcare provider can always see how I am doing	68.7	26.9	4.5
I find it annoying that I am being monitored with ALS H&C	1.5	11.9	86.6
***Feasibility***			
Using ALS H&C is quickly becoming a routine	62.6	23.9	13.4
I often forget to open and use the ALS H&C app	29.9	23.9	46.2
I am not able to make it a habit to use the ALS H&C app	17.9	26.9	55.2
Panel B – Healthcare providers			
***Acceptability***			
I am satisfied with the care concept ALS H&C	64.9	29.7	5.4
I am satisfied with the ALS H&C web portal	48.6	43.2	8.1
I feel comfortable using ALS H&C in my work	35.6	42.5	21.9
***Appropriateness/compatibility***			
An e-health care concept like ALS H&C fits with the way I work	70.9	26.4	2.8
An e-health care concept like ALS H&C fits with my organization	80.6	18.1	1.4
I find it important to be able to offer ALS H&C to patients	76.3	22.2	1.4
I like that with ALS H&C I can always see how a patient is doing	62.5	30.6	7.0
With ALS H&C I can better tailor the care to the needs of the patient	40.3	38.9	20.8
***Feasibility***			
Working with ALS H&C quickly became routine	23.6	54.2	22.2
I often forget to open and use the ALS H&C web portal	59.7	25.0	15.3
I am not able to make it a habit to work with the ALS H&C web portal	37.5	37.5	25.0
ALS H&C can be easily combined with my other work activities	47.2	34.7	18.1
Innovations such as ALS H&C reduce the workload of healthcare providers	13.9	54.2	32.0

*ALS* Amyotrophic lateral sclerosis, *ALS H&C* ALS Home monitoring & Coaching

The use of ALS H&C was also acceptable for the healthcare providers (Table 2, panel B); the majority was (very) satisfied (64.9%). On a scale from 1 (not satisfied at all) to 10 (very satisfied), healthcare providers rated the ALS H&C care concept with a 7.0 (median; IQR 1.0) and the platform with a 7.0 (median; IQR 1.8).

#### Appropriateness/compatibility

Most of the patients found ALS H&C to be an appropriate care concept (Table 2, panel A). Many patients (50.7%) reported that it meets their care needs and the majority (68.7%) liked the fact that a healthcare provider could always see how they were doing.

Most healthcare providers also reported that ALS H&C is an appropriate care concept, that it fits their way of working (70.9%) and within their organization (80.6%) (Table 2, panel B). And 40.3% thought that, with this concept, they could better tailor the care to the needs of the patients.

#### Feasibility

The results show that the (web) app is deemed suitable for everyday use by most patients (Table 2, panel A). For the majority (62.6%), the use of ALS H&C quickly became routine.

Healthcare providers were less positive about the feasibility of ALS H&C (Table 2, panel B). For the majority, working with ALS H&C did not become a routine quickly (76.4%). Many healthcare providers (54.2%) were not sure whether ALS H&C would result in a smaller workload or thought it would even lead to an increase (32.0%).

#### Fidelity

ALS H&C has been largely implemented as intended. To show the degree to which this was the case, any deviations from the original implementation plan as well as any changes to the original innovation were recorded and are listed in Table 3. Patient adherence to the agreed-upon monitoring assessments was lowest for the (daily) question on well-being (median 58.8%; IQR 67.3%) and high for weight and ALS-questionnaire (median 100%; IQR 16.7% and median 100%; IQR 0%, respectively).

**Table 3:** Fidelity issues

Main steps in the original implementation plan	Deviations from the original implementation plan
Implement ALS H&C in 10 teams in 3 cycles (3 teams in Cycle 1, 3 teams in Cycle 2, 4 teams in Cycle 3), so that learnings of the first Cycle could be incorporated in the next Cycle and so on.	Due to the COVID19-pandemic the original planning of the first implementation cycle had to be adjusted slightly. As a result, Cycle 1 was not finished yet before the start of Cycle 2 and therefore there were less opportunities to implement the learnings of the first cycle into the next.
Each participating center will form a project team, consisting of the physiatrist(s); two or three allied health professionals (i.e., one of them will become the healthcare coach, and one allied health professional will fulfill the role of knowledge broker); the manager; one scheduler; someone who will become the administrator of the platform and can provide technical assistance if necessary; one or two ALS patients; and one or two informal caregivers. The members of each project team will be involved in the implementation of ALS H&C within their organization.	Based on the original implementation plan, each team needed to have one healthcare provider who was willing to take on the role of knowledge broker. This had to be someone with an affinity for implementation, who knew the organization well, and who would be the driving force behind the realization of the action plans. In Team 6 the knowledge broker stopped after the first meeting due to personal reasons and the project team was not able to assign this role to another team member for a while. Team 5 did not include an informal caregiver in the project team.
Hold three preparatory meetings in which 1) ALS H&C will be introduced to the main stakeholders, 2) the target group and setting will be analyzed and the expected barriers/facilitators for implementation will be identified by the project team, and 3) the project teams develop action plans to address the expected barriers. These meetings will be held on site.	Due to the COVID19-pandemic physical visits were not possible. Instead, all meetings, except the first meeting with one team (Team 1), were held digitally via Zoom (videoconference). The third meeting of Team 5 (developing action plans) had to be rescheduled because there were too many no-shows at the official meeting. The healthcare professional that would take on the role of healthcare coach in Team 7 was not involved in the preparatory meetings (introduction, identifying barriers/facilitators, developing action plans) due to personal circumstances.
Each team will have three months for a pilot study with 5–10 patients to test and execute the implementation plans and to provide care with ALS H&C.	One team (Team 5) had some technical issues in the first month of the pilot phase and therefore it was decided to extend their pilot study with one month.
For each team there will be mid-term evaluations at 6 weeks (by phone/videoconference) and a final evaluation after three months (online surveys).	No deviations.
**Core elements of ALS H&C**	**Deviations from the original innovation**
ALS H&C consists of an application for patients that runs on smartphones and tablets, but can also be accessed through a computer. The application consists of a chat function for easy communication between patient and healthcare coach, a library where received information links can be saved, and three measurements: 1. A well-being question that can be answered with one of 10 smileys ranging from sad to happy and a written explanation/elaboration (optional) 2. Body weight 3. Functional status (ALSFRS questionnaire) The data will be passed on to a central server, where a healthcare professional can view it. The healthcare coach receives automated alerts whenever there is a significant change in well-being or body weight. The healthcare coach checks and follows up on the alerts and messages whenever necessary. They will monitor the data at least once a month with the monitor function on the platform. Data is shown in graphs and any significant changes are clearly indicated. The healthcare coach provides personalized feedback via a message in the app.	No deviations, but every team experienced some small temporary technical issues with the app/platform. These bugs were all resolved relatively quickly by the provider of the application.
One healthcare professional is assigned the role of healthcare coach. This person will perform the monitoring and will be the first point of contact for the patients. There is a low-threshold for patients to contact the healthcare coach, preferably via the chat in the app.	No deviations.
The patient is (as much as possible) in control.	No deviations.
The default frequencies for the measurements are daily for well-being, weekly for weight, and monthly for functioning, but the exact frequency of the measurements can be adjusted based on the wishes of the patient.	No deviations.
A healthcare protocol, which is based on the most recent treatment guidelines for physiotherapy, occupational therapy, speech therapy, etc., gives guidance to the healthcare coach for the monitoring, providing feedback and for sending information links. Participating centers are allowed and encouraged to slightly adjust the healthcare protocol to match their context, but without changing the core elements.	No deviations.
The healthcare coach provides at least once a month feedback to the patient regarding their measurements, even if there were no changes since the last monitoring.	Team 7 did not comply with/adhere to the healthcare protocol for the monitoring with regard to the monthly feedback. The protocol states patients should always receive feedback on their measurements (once a month) even when there are no changes in their situation since the last monitoring, but Team 7 did not always do this.
A fixed frequency of outpatient consultations at the clinic for all patients is not necessary anymore, because with ALS H&C the patient can be monitored continuously. Outpatient consultations can be planned based on the needs of the patient.	Most participating teams hold 3- to 4-monthly outpatient consultations with the physiatrist and other health care professionals of the ALS team to monitor disease progression of all patients. None of the teams felt comfortable letting go of this routine (completely) just yet.
Providing information is based on the patients’ needs.	No deviations.

^a^*ALS* Amyothrophic lateral sclerosis, *ALSFRS* Amyotrophic Lateral Sclerosis Functional Rating Scale, *ALS H&C* ALS Home monitoring & Coaching

#### Sustainability

Seven of the nine teams (78%) that completed the implementation process continued with ALS H&C after the pilot phase. Two teams (22%) decided to stop. The most important reported reason for not wanting to continue with ALS H&C for both teams, was the fact that the innovation was a stand-alone platform, and not integrated within their own electronic health record system.

### Technology acceptance and usability

#### Attitude (individual context)

In general, patients had a positive attitude to ALS H&C (Table 4, panel A). The majority of the patients (80.6%) reported that it was important to them that the option of home monitoring is offered to patients with ALS. Most patients liked using ALS H&C for their care (64.2%), thought it was helpful (59.7%), and did not experience the use of ALS H&C as a burden (76.1%).

**Table 4:** Technology acceptance and user experiences – Patients and Healthcare providers

	(Totally) Agree	Neutral	(Totally) Disagree
	%	%	%
**Panel A – Patients**			
***Attitude***			
I find the time it takes to answer the questions in the app is worth it	67.2	20.9	11.9
I find it important that the option of home monitoring is offered to patients with ALS	80.6	13.4	6.0
The usability of the app is an important determinant in my choice to use the app or not	80.6	16.4	3.0
The attractiveness of the app is an important determinant in my choice to use the app or not	32.9	40.3	26.9
I think it is good to use ALS H&C for my care	79.1	16.4	4.5
I like using ALS H&C for my care	64.2	35.8	0.0
I find the use of ALS H&C in my care helpful for my care	59.7	31.3	9.0
I find the use of ALS H&C in my care burdensome	6.0	17.9	76.1
I find the use of ALS H&C in my care time consuming	7.5	16.4	76.1
I think the care I receive with ALS H&C is better than the care I received without this care concept	35.8	41.8	22.4
	**Yes**	**No, just right**	**No**
	**%**	**%**	**%**
I find the frequency of the well-being measurements too high	52.2	47.8	0.0
I find the frequency of the weight measurements too high	16.4	83.6	0.0
I find the frequency of the ALSFRS-R measurements too high	4.5	95.5	0.0
I find the frequency of the feedback of my healthcare coach on my measurements too low	16.4	83.6	0.0
***Perceived (Technology) Control***			
I can decide for myself whether to use ALS H&C	82.1	4.5	13.5
I experience more control over my care with ALS H&C	55.5	29.9	14.9
	**Yes**	**Sometimes**	**No**
	**%**	**%**	**%**
I can use the ALS H&C app without assistance from others	89.6	4.5	6.0
I let other healthcare providers (e.g., my GP) have a look in the app	28.4	6.0	65.7
	**(Totally) Agree**	**Neutral**	**(Totally) Disagree**
***Perceived usefulness***	**%**	**%**	**%**
I find the app useful	76.1	17.9	5.6
I find the app insightful	83.6	11.9	4.5
I find the measurements informative	70.2	22.4	7.5
I find the measurements insightful	71.6	22.4	6.0
I find the messages/feedback I receive from the healthcare coach in the app easy to understand	80.6	19.4	0.0
I find the messages/feedback I receive from the healthcare coach in the app informative	70.2	29.9	0.0
I find the messages/feedback I receive from the healthcare coach in the app useful	71.7	28.4	0.0
I find the e-mails I receive to remind me I need to complete the measurements useful	79.1	14.9	6.0
***Perceived ease of use***			
I found it easy to install the app	62.0	22.5	15.5
I find logging into the app difficult	9.8	22.5	67.6
I find logging into the app is slow	29.6	32.4	38.0
I find it easy to operate the app	76.0	19.7	4.2
I find it easy to learn how to operate the app	83.1	14.1	2.8
I find the app slow	29.6	42.3	28.2
	**Yes**	**Sometimes**	**No**
	**%**	**%**	**%**
I was able to answer the well-being question with the smileys in the app	91.4	2.9	5.7
I was able to add additional explanations to the well-being question in the app	92.9	1.4	5.7
I was able to complete the weight measurements in the app	93.0	0.0	7.0
I was able to complete the ALSFRS-R questionnaire in the app	98.6	0.0	1.4
I was able to read the feedback and messages from the healthcare coach in the app	87.3	5.6	7.0
I was able to send messages to the healthcare coach in the app	88.6	1.4	10.0
I was able to open the information links the healthcare coach added to the app	89.9	0.0	10.1
I have had problems with logging into the app	23.9	N.A.	76.1
	**(Totally) Agree**	**Neutral**	**(Totally) Disagree**
***Peer influence***			
People who are important to me think that I should use ALS H&C	37.3	43.3	19.4
My physiatrist(s)/ALS care team find it important that I use ALS H&C in my care	53.7	40.3	6.0
***Intention***			
I intent to keep using ALS H&C in the next six months	88.0	9.0	3.0
I would recommend ALS H&C to other patients with ALS	79.1	17.9	3.0
**Panel B – Healthcare providers**			
***Attitude***			
I find it a good idea to offer home monitoring as part of regular care to patients with ALS	80.8	13.7	5.5
ALS H&C is beneficial to my patient care and management	46.5	43.8	9.6
I think it is good to use ALS H&C in my work	64.4	32.9	2.7
I like using ALS H&C in my work	31.5	65.8	2.7
I find the use of ALS H&C in my work helpful for the care I provide	58.9	28.8	12.3
I find the use of ALS H&C in my work burdensome	23.3	39.7	37.0
I find the use of ALS H&C in my work time consuming	27.4	49.3	23.3
I think the care I provide with ALS H&C is better than the care without ALS H&C	32.9	49.3	17.8
***Perceived (Technology) Control***			
I would have the ability to use ALS H&C in my patient care and – management	75.4	16.4	8.2
I can decide for myself whether to use ALS H&C in my work	45.2	24.7	30.2
I do not have enough knowledge to use ALS H&C properly	9.6	24.7	65.8
I have enough skills to use ALS H&C properly	60.3	23.3	16.4
I can use the ALS H&C web portal without assistance from others	76.4 (yes)	16.7 (sometimes)	6.9 (no)
***Perceived usefulness***			
ALS H&C cannot improve my patient care and management	12.3	31.5	56.1
ALS H&C cannot enhance my effectiveness in patient care and management	17.8	41.1	41.1
ALS H&C cannot make my job any easier	17.8	31.5	50.6
I find ALS H&C not useful for my patient care and management	5.5	24.7	69.9
ALS H&C is an improvement of the regular ALS care	58.9	34.2	6.8
E-health has no added value for ALS care	2.7	15.1	82.2
I find being able to view the monitoring data of patients on the ALS H&C web portal insightful	71.3	23.3	5.5
I find being able to view the monitoring data of patients on the ALS H&C web portal informative	73.9	21.9	4.1
I find being able to view the monitoring data of patients on the ALS H&C web portal useful	69.8	27.4	2.8
***Perceived ease of use (only health care coaches)***			
I find it easy to create accounts for patients	85.7	14.3	0.0
I find it easy to set up measurement trajectories for patients	85.7	14.3	0.0
I find it easy to communicate with patients via the chat function of ALS H&C	92.8	0.0	7.1
I find it useful to communicate with patients via the chat function of ALS H&C	78.6	0.0	21.4
I find it easy to send information links to patients via the ALS H&C web portal	64.3	14.3	21.4
I find it useful to send information links to patients via the ALS H&C web portal	50.0	21.4	28.6
I find it easy to switch between the different tabs in the ALS H&C web portal	100.0	0.0	0.0
I find it confusing to switch between the different tabs in the ALS H&C web portal	7.1	14.3	78.6
Finding a patient in the ALS H&C web portal is very easy	28.5	35.7	35.7
Handling new messages in the ALS H&C web portal is easy	78.6	7.1	14.3
Handling new alerts in the ALS H&C web portal is easy	64.3	21.4	14.3
Setting up additional measurements for patients is easy	85.7	14.3	0.0
Adjusting the frequency of measurements for patients is easy	71.4	14.3	14.2
***Perceived ease of use***			
I found it difficult to learn how to work with ALS H&C	8.0	41.3	50.7
I find logging into the ALS H&C web portal difficult	13.5	45.9	40.6
I find logging into the ALS H&C web portal slow	16.3	48.6	35.2
I have had problems with logging into the app	37.8 (yes)	N.A.	62.2 (no)
I find navigating the ALS H&C web portal slow	8.1	58.1	33.8
***Peer influence***			
People who influence my clinical behavior think that I should use ALS H&C in my work	18.1	52.8	29.2
It is expected of me that I use ALS H&C in my work	41.7	33.3	25.0
I experience pressure from others to use ALS H&C in my work	9.7	38.9	51.4
***Intention***			
I intent to keep using ALS H&C in the next six months	66.7	27.8	5.6
I would recommend ALS H&C to colleagues of other ALS care teams	55.5	36.1	8.3

*ALS* Amyotrophic lateral sclerosis, *ALS H&C* ALS Home monitoring & Coaching, *GP* General practitioner

The attitude of the healthcare providers to ALS H&C was also mainly positive (Table 4, panel B). The majority (80.8%) reported that it is a good idea to offer home monitoring as part of the regular care to patients with ALS. Most healthcare providers (58.9%) reported that they think ALS H&C is helpful in providing care to their patients.

#### Perceived (technology) control (individual context)

As shown in Table 4 (panel A), most patients (82.1%) perceived that they can decide for themselves whether to use ALS H&C. The majority (55.5%) indicated that with ALS H&C they experienced more control over their care. Most patients (89.6%) were able to use the (web) app without support from someone else.

Most healthcare providers (75.4%) indicated that they could use ALS H&C for their patient care and – management (Table 4, panel B). The majority reported that they had sufficient knowledge (65.8%) and skills (60.3%) to work with ALS H&C. The majority (76.4%) was able to use the platform without assistance from others.

#### Perceived usefulness (technological context)

Patients were mainly positive regarding the perceived usefulness of ALS H&C (Table 4, panel A). The majority perceived the (web) app as useful (76.1%) and insightful (83.6%). Most patients reported that the measurements were informative (70.2%) and insightful (71.6%); they also perceived the feedback and messages from the healthcare coach to be informative (70.2%) and easy to understand (80.6%).

The healthcare providers perceived ALS H&C as useful (Table 4, panel B). The majority reported that e-health adds value to (82.2%) and is an improvement of regular ALS care (58.9%). Most healthcare providers indicated that being able to monitor the patients in the web portal is useful (69.8%), insightful (71.3%) and informative (73.9%).

#### Perceived ease of use and usability (technological context)

Patients were mainly positive about the perceived ease of use of several aspects of the (web) app (Table 4, panel A). For example, the majority indicated that it was easy both to log in (67.6%) and that to learn how to use the web (app) (83.1%).

Healthcare coaches (*n* = 14) were very positive about the perceived ease of use of several aspects of the web portal (Table 4, panel B). Most indicated that it was easy to create accounts (85.7%), to set up the measurement trajectories (85.7%) and to adjust them when necessary (71.4%). Communicating with patients via the chat function was considered as easy (92.8%) and useful (78.6%). The overall usability of the web-portal according to the healthcare coaches was average, while other healthcare providers considered the usability below-average. Mean (SD) SUS scores were 68.9 (10.7) and 58.8 (11.3), respectively.

The other healthcare providers (*n* = 62) were fairly positive about the perceived ease of use of several aspects of the web portal (Table 4, panel B). For 50.7% of the healthcare providers, it was easy to learn to work with the web portal and most of them (86.5%) indicated that it was not difficult to log in. However, 37.8% reported that they had occasional problems with login (forgotten password, typos in the username/password or a technical bug in the system).

#### Peer influence (implementation context)

Peer influence did not play a large role for most patients (Table 4, panel A). Only 37.3% of the patients reported that people who are important to them think that they should use ALS H&C. Many patients (53.7%) reported that their physiatrist(s) and/or ALS care team consider it important that they used ALS H&C.

Nor did peer influence play a large role for most healthcare providers (Table 4, panel B). A minority of the healthcare providers (18.1%) thought that people who influence their clinical behavior think that they should use ALS H&C, but 51.4% felt no pressure from others to actually use it.

#### Advantages and disadvantages

The advantages of ALS H&C most often mentioned by patients were: 1) the feeling of being more in control of their own care, 2) easy communication with the healthcare coach and ALS care team, 3) that they had more insight into their daily functioning and disease progression and that their healthcare team also had more insight, 4) that they did not have to go to the rehabilitation centre as often, 5) that the healthcare team could act immediately whenever there was a change in their situation, and 6) that the care could be more personalized and organized based on their needs.

The main disadvantages of ALS H&C, according to patients, were 1) app slowness, 2) occasional technical problems with logging in, 3) questions and answer categories not being specific enough, 4) the daily well-being question was too general, 5) the high frequency of being asked about well-being 6) lack of notifications for new messages, and 7) being confronted with (progression of) the disease.

The main advantages of ALS H&C for the healthcare providers were 1) that they had more insight into the daily functioning and disease progression of the patients, 2) that they could identify problems earlier, and, therefore, intervene earlier, 3) that they could easily communicate with the patients, also with patients who did not come to the rehabilitation centre very often.

The disadvantages of working with ALS H&C most often mentioned by the healthcare providers were 1) the technical problems they encountered, 2) the fact that the platform was not integrated with their own electronic patient records, 3) that it was time consuming, especially at the start, 4) that the measurements were not specific enough (e.g. lack of pulmonary function tests, no questions on micturition and defecation) and not always tailored to the specific situation of the patient, 5) and having difficulties with “letting go of control” and trusting their colleagues to update and notify them when necessary, and doubt about the patients’ abilities to complete the ALSFRS-R truthfully.

#### Intention

The intention to continue using ALS H&C and to recommend it to others was high, especially for patients (Table 4, panel A). The majority of the patients (88.0%) intended to keep using ALS H&C for the next 6 months, and 79.1% would recommend that other ALS patients use it.

The intention of healthcare providers to continue using and to recommend ALS H&C was lower than patients (Table 4, panel B). The majority of the healthcare providers (66.7%) intended to keep using ALS H&C for the next 6 months, and 55.5% reported that they would recommend other colleagues use it.

### Differences in implementation outcomes between teams with sustainable and unsustainable implementation

Although there were no notable differences in expected barriers or action plans, there were several differences in the implementation outcomes between the seven teams where the implementation was sustainable (continuation after the pilot phase) and where the implementation was not sustainable (discontinuation after the pilot phase – 2 teams).

One team that discontinued ALS H&C had four *fidelity* issues. The first fidelity issue was the fact that no informal caregiver was included in the project team. The second was the extension of the pilot phase due to technical issues at the start. The third was the fact that the third meeting (developing action plans) had to be rescheduled because too many team members did not show up, which may have been a sign of commitment issues. This was in line with the fact that the kick-off meeting in which the project group was going to inform and motivate the rest of the healthcare team was not well attended by their colleagues (field notes). Furthermore, field notes also revealed that there was some hesitance from the start among some of the healthcare providers about working with ALS H&C, and that there may have been some communication/trust issues within the care team. The fourth fidelity issue was that the fixed frequency of outpatient consultations remained (largely) unchanged. The other team with unsustainable implementation had three fidelity issues: (1) due to personal circumstances, the primary healthcare coach did not attend the preparatory meetings with the project team, (2) the fixed frequency of outpatient consultations remained (largely) unchanged, and (3) they did not completely follow the healthcare protocol for the monthly monitoring. Compared to healthcare providers of the teams with sustainable implementation (*n* = 59), the healthcare providers of the 2 teams that discontinued ALS H&C (*n* = 15) scored lower on *usability* (mean [SD] SUS = 53,3 [11.7] versus 62,6 [1, 11]). Numeric ratings of *acceptability* were somewhat lower in teams with unsustainable implementation compared to healthcare providers of the teams with sustainable implementation (mean rating of the ALS H&C care concept was 6.7 [SD 1.3] versus 7.0 [SD 1.4]. For the web portal the mean [SD] scores were 5.9 [SD 1.9] and 6.8 [SD 1.4] respectively. Results suggest that the healthcare providers of the teams that discontinued ALS H&C were less positive than the other healthcare providers about *perceived usefulness, attitude, appropriateness, feasibility*, and *intention,* but due to the small sample size this could not be tested statistically.

Despite the aforementioned lower scores on healthcare providers’ implementation outcomes and fidelity issues, there were no significant differences in acceptability of patients between the teams that discontinued ALS H&C (*n* = 14) and teams with sustainable implementation (*n* = 53). Patients in the two teams that discontinued ALS H&C rated the ALS H&C care concept on average with a 7.1 [SD 1.5] versus 7.7 [1.4] by patients in other teams. Patients mean scores for the (web) app were 6.9 [SD 1.5] and 7.3 [SD 1.5] respectively. Although it could not be tested due to the small sample size, patients in one of the two teams that discontinued ALS H&C appeared to be less positive than the other patients regarding *perceived ease of use, acceptability* and *attitude*.

## Discussion

The present study demonstrates that a participatory action research approach supported by theoretical approaches used in implementation sciences is a promising method for implementing e-health innovations in multidisciplinary rehabilitation settings. This combined approach has been used successfully in other research areas as well [26, 27], but it had not yet been used for implementing an e-health innovation in a multidisciplinary rehabilitation setting. In this study, the approach resulted in mainly positive implementation outcomes, a good technology acceptance and fairly good user experiences with the e-health innovation ALS H&C. The implementation of ALS H&C was sustainable in seven out of nine rehabilitation settings that completed the implementation process. Feasibility, usability, and fidelity issues played an important role in implementation failure.

Overall, patients were positive about the care concept ALS H&C and about using the application. The overall adoption rate was high and patients found ALS H&C an acceptable, appropriate, and feasible care concept. The technology acceptance was also high, with patients scoring positively on determinants related to the individual, technological and implementation context [31]. Altogether, this has led to a strong intention to continue using ALS H&C in the future.

In general, healthcare providers’ implementation outcomes, technology acceptance and user experiences were also positive, but healthcare providers were less positive about the feasibility and usability of ALS H&C. This seems to have reflected negatively on their intention to continue using ALS H&C. Overall, only 66.7% intended to continue to use ALS H&C in the next 6 months. In particular healthcare providers who did not use the platform on a regular basis found it to be not user-friendly. It is understandable that if a platform is not user-friendly, it is difficult to make a habit of using it (feasibility); therefore, the intention to continue using it is low. This suggests that the usability of a platform is an important factor for the success of the implementation, which is in line with previous research [35–37]. In contrast to the other healthcare providers, the healthcare coaches were positive about the usability of the platform. They received an extensive training in monitoring patients and using the platform. During the pilot phase they gained more experience with ALS H&C compared to other healthcare providers who were not specifically trained and who only received the platform instruction manual. Training all healthcare providers of the multidisciplinary team who are involved in the care of patients with ALS is recommended as an additional implementation strategy. The learnability of e-health is known to be a factor of usability; training sessions can reduce the time users need to be able to work with the e-health application [37].

At team-level, it can be concluded that ALS H&C was sustainably implemented by 78% of the participating teams (*n* = 7). Two teams discontinued ALS H&C after the pilot phase. The main (self-reported) reason for discontinuation was the lack of integration with their own electronic patient records system. Although lack of system interoperability was a foreseen barrier for professional acceptability [38], integrating ALS H&C into the different electronic patient records systems is a technical and costly challenge that could not be solved within the allocated budget and time available. Technical integration is possible and available in one centre (UMC Utrecht) where it has been shown to enhance the health information exchange and thereby the ability of all healthcare providers to act more proactively [9]. Integrating the platform with the electronic patient records system is recommended as an additional implementation strategy.

Comparing the implementation outcomes between the teams with sustainable and unsustainable implementation illustrated the importance of fidelity when implementing an e-health innovation. The fidelity issues in the two teams with unsustainable implementation may have had a knock-on effect on some of the other implementation outcomes. Although the comparative exploration was based on small samples the findings suggests that fidelity issues mainly impacted on implementation outcomes of the healthcare providers. There are two reasons why this is not completely surprising. First, ALS H&C was mainly developed for the benefit of the patients, and less so for the benefit of healthcare providers. When implemented as intended, patients are likely to experience mainly the advantages of this new care concept (e.g., more control, easy communication, less traveling, more insight), while healthcare providers themselves may also experience some disadvantages, such as the extra time investment at the start when learning to work with ALS H&C or changing work routines. Secondly, the implementation of ALS H&C constitutes more fundamental changes for healthcare providers than for patients. For example, healthcare providers need to adjust their work routines and behavior. Such organizational and behavioral changes are often difficult and take time and effort, even after the official end of an implementation project [39, 40]. Research has shown that it can take up to 254 (average 66) days of fairly consistent repetition before a new behavior becomes a habit [39]. Moreover, practicing the new behavior as often as possible at the start has a stronger impact on habit formation processes than practicing it later on in the process [39]. Therefore, it is recommended that every healthcare provider involved in the care of patients with ALS, practices and starts using the platform as soon as possible and as often as possible, instead of solely relying on one person for monitoring the platform and providing feedback to their colleagues.

When taking a closer look at the teams where implementation was not sustainable, a few additional lessons can be learned. It is important to pay extra attention to potential communication/trust issues within a team, and the attitude to the innovation of every team member who will be affected by the implementation. This should be done before the start of an implementation project. If skepticism or resistance to the innovation is present in certain individuals, it is important to spend extra time and effort to take away their concerns and explicitly also involve these people in the project team. In this way, the identification of barriers and facilitators will truly reflect all points of view, and only then can appropriate action plans, leading to successful implementation, be developed. In addition, it is imperative that the persons who will be affected most by the implementation are involved from the start. This will increase the likelihood that protocols will be followed as intended. The findings of this study suggest that the involvement of the healthcare coach has a large impact on the implementation success. Healthcare coaches have a pivotal position in this care concept; in relation to both the patients and their colleagues. Therefore, it is paramount to choose the healthcare coach wisely. We recommend working out a very detailed profile and list of necessary requirements for selecting the best person for the most crucial role within a new care concept. A project ‘champion’ (a person who takes responsibility for and is the driving force in the implementation) is indispensable to ensure the plans are actually carried out. According to literature, project ‘champions’ have a positive effect on implementation outcomes and are often considered as a key factor for implementation success [41].

### Limitations

This study was performed in the Dutch context of rehabilitation settings with multidisciplinary ALS teams that are part of the ALS Care Network. All certified teams (*n* = 35) are supported by the expert centre for ALS through sharing best practice, guideline development and implementation, continuous learning (training and e-learnings) and patient education. Furthermore, in the Netherlands internet access is very high with 97% of the general Dutch population having access and 88% daily use of internet [42]. These contextual factors may limit the generalizability of the implementation outcomes to other contexts in ALS care. However, the participatory action research approach, the mentored implementation, and evaluation likely have broad applicability.

## Conclusions

This study has shown that the e-health innovation ALS H&C can be successfully implemented in different multidisciplinary rehabilitation settings. The successful implementation strategy consisted of a participatory action research approach, leveraging frameworks and process models from the field of implementation science. Feasibility, usability and fidelity played an important role in implementation failure. Therefore, to improve implementation success, a number of additional implementation strategies are recommended:

- Sufficient training of healthcare providers on how to use the innovation.
- Integration of the e-health web portal with the local electronic health record system.
- Ensure high fidelity by not changing the core elements of the implementation nor of the innovation .

Additional lessons from this study that can be useful for other implementation scientists and – practitioners aiming to implement an e-health innovation in a multidisciplinary setting:

- Pay special attention to potential communication/trust issues within a team and the teams’ attitude towards the innovation at the start of an implementation project;
- Use positive experiences of patients to convince the healthcare providers of the value of the innovation;
- Ensure at least one person is selected as project champion who is responsible for and who is the driving force behind the realization of action plans;
- Ensure that the right people are selected for the crucial positions in the team and 5) ensure that they are involved right from the start.

## Supplementary Information

**Additional file 1: Supplementary Figure 1**.

## Data Availability

The datasets used and/or analyzed during the current study are available from the corresponding author on reasonable request.

## Abbreviations

ALS: Amyotrophic lateral sclerosis
ALSFRS-R: Amyotrophic Lateral Sclerosis Functional Rating Scale-Revised
ALS H&C: ALS Home monitoring & Coaching
IQR: Interquartile range
N: Sample size
PLS: Primary lateral sclerosis
PMA: Progressive spinal muscular atrophy
SD: Standard deviation
SUS: System Usability Scale
