# Supplementary figures and images for: Evaluation of the nation-wide implementation of ALS home monitoring & coaching: an e-health innovation for personalized care for patients with motor neuron disease
Source: BMC Health Serv Res. 2022 Nov 22;22:1389. doi: 10.1186/s12913-022-08724-6 (PMC9682770; doi:10.1186/s12913-022-08724-6)

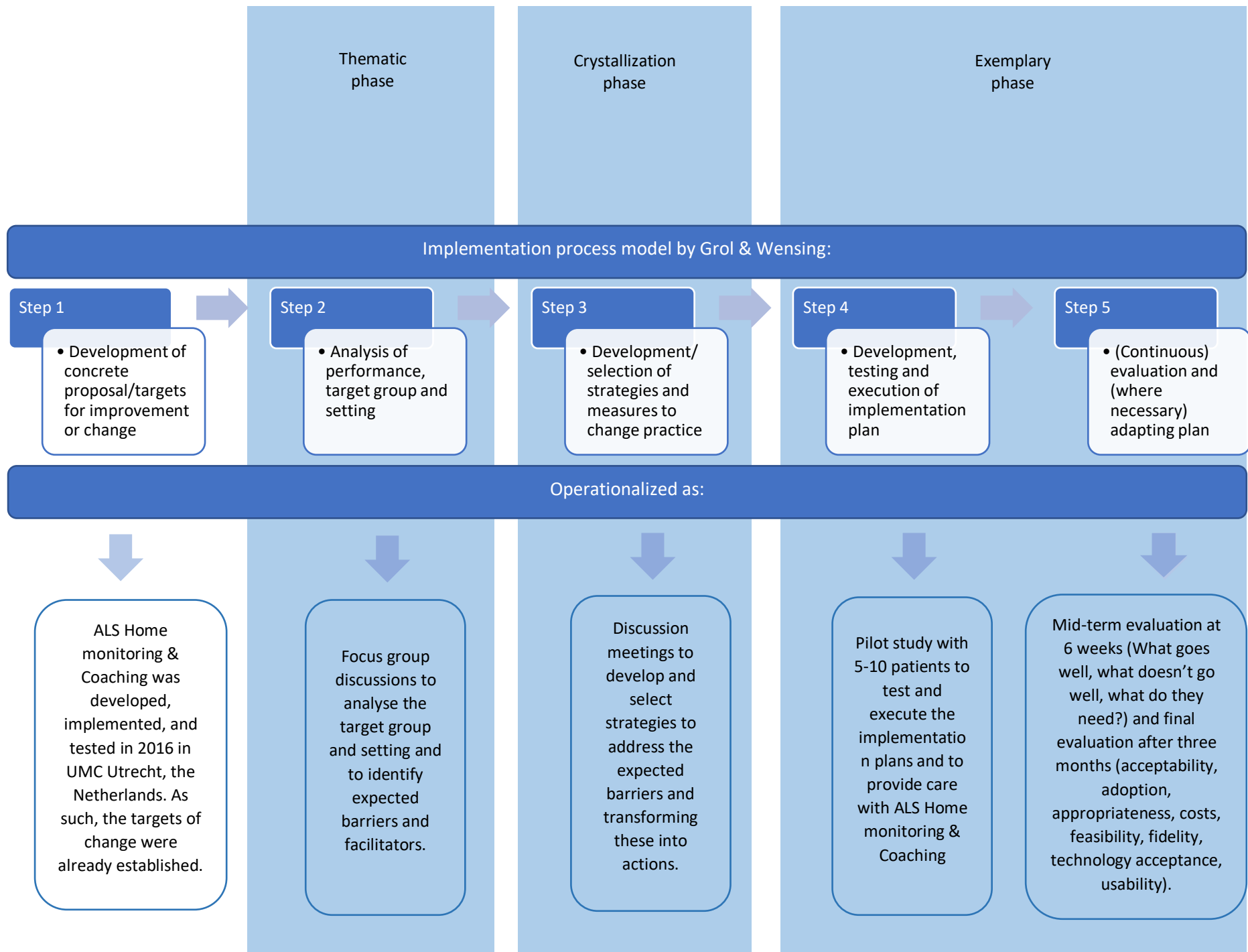

Supplement: Supplementary file 1 — Additional file 1: Supplementary Figure 1. [file 12913_2022_8724_MOESM1_ESM.pdf]
